# Malignant uterine PEComa: a narrative literature review and challenging case report

**DOI:** 10.1007/s00432-026-06487-9

**Published:** 2026-05-20

**Authors:** Mauro Francesco Pio Maiorano, Joana Sorino, Mario Della Mura, Doriana Di Nanni, Gerardo Cazzato, Alessandro Rizzo, Stella D’Oronzo, Marco Cerbone, Ettore Cicinelli, Marco Marinaccio

**Affiliations:** 1 Unit of Obstetrics and Gynecology, Department of Interdisciplinary Medicine (DIM), University of Bari “Aldo Moro,” Policlinico of Bari, Bari, BA Italy; 2 Section of Molecular Pathology, Department of Precision and Regenerative Medicine and Ionian Area (DiMePRe-J), University of Bari “Aldo Moro”, Bari, 70124 Italy; 3 TDM, IRCCS Istituto Tumori “Giovanni Paolo II”, Viale Orazio Flacco 65, Bari, 70124 Italy; 4 Interdisciplinary Department of Medicine, University of Bari Aldo Moro, Bari, Italy; 5 Medicine and Surgery Department, LUM Degennaro University, Casamassima, Bari, Italy

**Keywords:** Perivascular epithelioid cell tumor, PEComa, Uterine neoplasm, Surgical excision, Tuberous sclerosis complex, Mammalian target of rapamycin inhibitors

## Abstract

Uterine perivascular epithelioid cell tumors (PEComas) are exceptional mesenchymal neoplasms of the gynecologic tract that often mimic common uterine lesions and lack pathognomonic clinical or radiologic features. We report a malignant uterine PEComa in a 44-year-old woman presenting with abnormal uterine bleeding and pelvic pain. Preoperative imaging suggested a fibroid, but definitive diagnosis followed hysterectomy: in fact, histopathology showed epithelioid cells arranged around a rich vasculature with high-grade cytologic atypia, brisk mitotic activity, and necrosis, all features consistent with malignant behavior. Subsequent immunophenotyping demonstrated co-expression of melanocytic and smooth-muscle markers. The disease was organ-confined; no adjuvant therapy was administered, and the patient remains disease-free at 24 months under structured surveillance. To contextualize this case, we conducted a narrative review of the literature through October 2025, synthesizing epidemiology, pathogenesis, diagnosis, histology, treatment, and outcomes of uterine PEComas. Evidence supports complete surgical excision as the cornerstone of management for localized disease. For advanced or recurrent tumors, inhibitors of the mammalian target of rapamycin (mTOR) represent a rational option given frequent dysregulation of this pathway. Overall, malignant uterine PEComa requires multidisciplinary evaluation, pathology-driven diagnosis, and long-term follow-up; accumulating case-based evidence is refining risk stratification and informing the selective use of targeted therapies.

## Introduction

PEComas of the gynecologic tract are exceedingly rare mesenchymal neoplasms composed of perivascular epithelioid cells (PECs) associated with blood vessel walls and characterized by a distinctive myomelanocytic phenotype, defined by the co-expression of melanocytic markers (HMB-45, Melan-A) and smooth muscle markers (SMA, desmin) (Fink et al. 2004). While they can arise in various anatomical sites, approximately 25% occur in the gynecologic tract, with the uterine corpus being the most common location (75%) (Musella et al. 2015). Current knowledge of this entity has been derived primarily from case reports and small case series. Fewer than 110 uterine PEComa cases have been documented in the literature worldwide from their first description in 1990s, underscoring their extreme rarity (Oliver-Perez et al. 2025). Among these, only a minority have exhibited malignant behavior, which has been associated with specific clinicopathological features (Musella et al. 2015, Hu et al. 2024). Patients span a wide age range (reported from 9 to 79 years), with a peak incidence in the fourth decade of life (Musella et al. 2015, Jiang et al. 2023). Most cases are sporadic; however, 8–10% of PEComas are associated with tuberous sclerosis complex (TSC) (Jiang et al. 2023, Conlon et al. 2015, Fadare 2008). Clinical presentation is nonspecific, including abnormal uterine bleeding and pelvic or abdominal pain, and rarely uterine rupture or hemoperitoneum during pregnancy (Musella et al. 2015, Schoolmeester et al. 2014, Nguyen et al. 2020). As PEComas lack specific clinical and radiologic features, the diagnosis is challenging and can definitively be achieved only after pathological evaluation (Wang et al. 2024, Fontana et al. 2021). In particular, they must be distinguished from leiomyomas and other uterine mesenchymal neoplasms due to overlapping radiologic characteristics (Phillips et al. 2016). In summary, uterine PEComas are rare and diagnostically challenging tumors that often mimic more common uterine lesions. Their rarity and heterogeneity have precluded robust epidemiologic studies or standardized guidelines (Hu et al. 2024). Malignant behavior cannot be predicted with certainty preoperatively, making thorough pathologic assessment and judicious application of criteria for guiding management. We report a challenging case of malignant uterine PEComa and narratively review the relevant literature regarding epidemiology, diagnosis, and management of this rare entity.

## Methods

This manuscript combines a narrative literature review with a single-patient case report, structured in accordance with accepted recommendations for rare disease documentation.

The narrative review was conducted to provide context and highlight key clinical and pathological features of uterine PEComas. Literature searches were performed using PubMed/MEDLINE, Scopus, and Google Scholar through October 2025, using combinations of the keywords: “PEComa,” “uterine PEComa,” “malignant PEComa,” “gynecologic mesenchymal tumors,” “TSC,” and “mTOR inhibitors.” Articles selected included case reports, case series, and relevant narrative and systematic reviews. Preference was given to English-language publications with indexed references and available abstracts. Relevant findings from the literature were organized thematically: epidemiology, pathogenesis, diagnosis, histology, treatment and future direction, and outcomes, and integrated into the results and discussion sections.

The case was reported in accordance with the CARE (CAse REport) guidelines, ensuring inclusion of critical elements such as patient presentation, diagnostic reasoning, therapeutic interventions, follow-up, and patient perspective (Gagnier et al. 2013). Written informed consent was obtained from the patient for publication of this anonymized case, including associated imaging and pathology data (Table 1).

**Table 1:** CARE checklist for case reports

CARE item	Description
Patient Information	Age, sex, and presenting symptoms – included
Clinical Findings	Physical exam, imaging, and pathology findings – included
Diagnostic Assessment	Imaging, histology, immunohistochemistry – included
Therapeutic Intervention	Surgery, chemotherapy – included
Follow-up and Outcomes	24-month surveillance, disease-free – included
Discussion of Relevant Literature	Included
Informed Consent	Patient consent obtained – included

## Results and case presentation

**Fig. 1:**
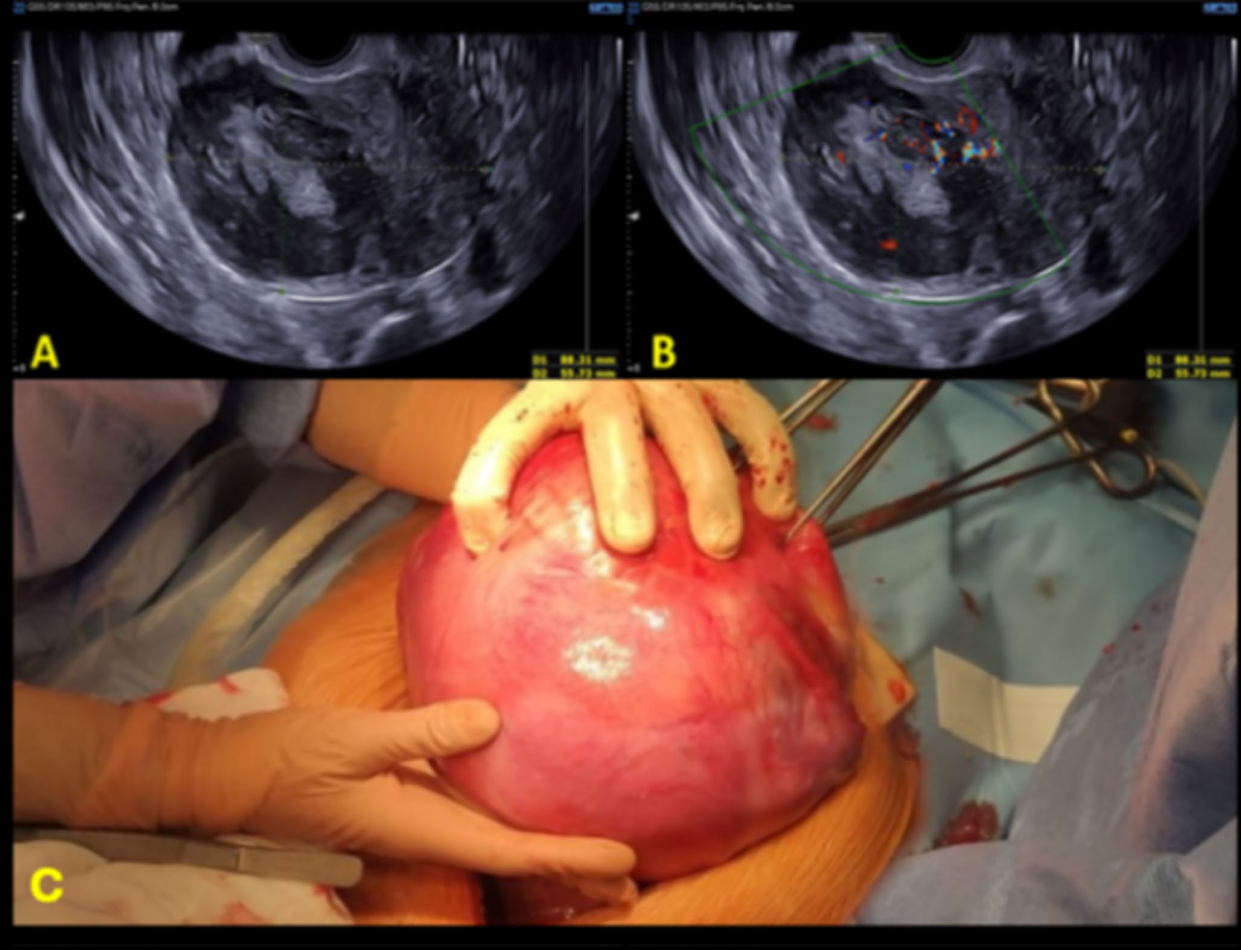
Transvaginal ultrasound shows a single large lesion (≈ 9 cm) with well-defined margins and a heterogeneous echotexture due to the presence of hypoechoic areas, likely of cystic-degenerative nature (**A**), with visible vascularization on Doppler (**B**). During surgery, a fleshy mass was enucleated (**C**)

The patient in our case is a middle-aged woman of 44 years who presented with a 6-month history of vaginal bleeding and pelvic pain, symptoms that are common but non-specific for uterine PEComas (Musella et al. 2015, Oliver-Perez et al. 2025, Schoolmeester et al. 2014, Nguyen et al. 2020, Giannella et al. 2020). On pelvic examination, an enlarged uterus or mass was noted, raising suspicion for a uterine neoplasm. Indeed, abnormal uterine bleeding is the most frequent presenting symptom of uterine PEComa (around 40% of cases), often accompanied by pain (25–30% of cases) (Hu et al. 2024, Cao and Huang 2022). The patient’s initial symptoms and exam findings were indistinguishable from those of a uterine fibroid or other uterine tumors, which reflects a well-documented diagnostic pitfall with these rare tumors (Hu et al. 2024, Cao and Huang 2022). There were no stigmata of TSC (such as skin lesions or a history of renal angiomyolipomas) in this patient, and no known family history of TSC, making a sporadic PEComa more likely (Conlon et al. 2015, Schoolmeester et al. 2014, Wang et al. 2024). Pelvic imaging was obtained as part of the initial workup. Transvaginal ultrasound revealed a uterine mass that was presumed to be a leiomyoma (fibroid) due to its solid appearance and location (Fig. 1). This misdiagnosis is typical: uterine PEComas on ultrasound usually appear as a well-defined, solid mass with heterogeneous echotexture and a rich internal blood flow on Doppler, findings that overlap significantly with benign fibroids or uterine sarcomas (Oliver-Perez et al. 2025, Wang et al. 2024, Fontana et al. 2021, Phillips et al. 2016, Giannella et al. 2020, Yu et al. 2024). In our case, Doppler ultrasound showed abundant central vascularity in the mass, which is consistent with PEComa’s hypervascular nature, but at the time it was attributed to a vascular leiomyoma (Oliver-Perez et al. 2025, Fontana et al. 2021). No calcifications or specific shadowing were noted on ultrasound, which somewhat argued against a calcified fibroid, but is still not diagnostic (Oliver-Perez et al. 2025, Wang et al. 2024, Fontana et al. 2021, Phillips et al. 2016, Giannella et al. 2020, Yu et al. 2024). Given the atypical features, further imaging was performed. Magnetic resonance imaging (MRI) demonstrated a ~ 10 cm uterine lesion with predominantly intermediate T1 signal and high T2 signal, with areas of internal hemorrhage. These MRI characteristics are described in PEComa but are also seen in degenerating fibroids and sarcomas (Phillips et al. 2016, Giannella et al. 2020, Yu et al. 2024). Importantly, no definitive fat signal was observed within the tumor on MRI; while intratumoral fat can sometimes suggest a PEComa, its absence does not rule it out. The imaging did not show gross extrauterine spread. In summary, the preoperative imaging studies were unable to conclusively distinguish this rare tumor from more common uterine lesions, a limitation echoed in the literature (Oliver-Perez et al. 2025, Wang et al. 2024, Fontana et al. 2021, Phillips et al. 2016, Giannella et al. 2020, Yu et al. 2024). Consequently, the working diagnosis remained a uterine fibroid or possibly a leiomyosarcoma, and surgical management was pursued accordingly. The patient underwent a hysterectomy (with bilateral salpingo-oophorectomy), which is the typical surgical approach for a suspicious uterine mass in a woman who has completed childbearing. At surgery, the uterine tumor was found to be a fleshy, uterine mass of 9 cm. There were no obvious macroscopic metastases. The resected specimen revealed a tumor centered in the uterine corpus. On gross examination, the mass was fleshy, tan-pink with areas of hemorrhage and necrosis, features that raised concern for a malignant process (Fig. 1).

**Fig. 2:**
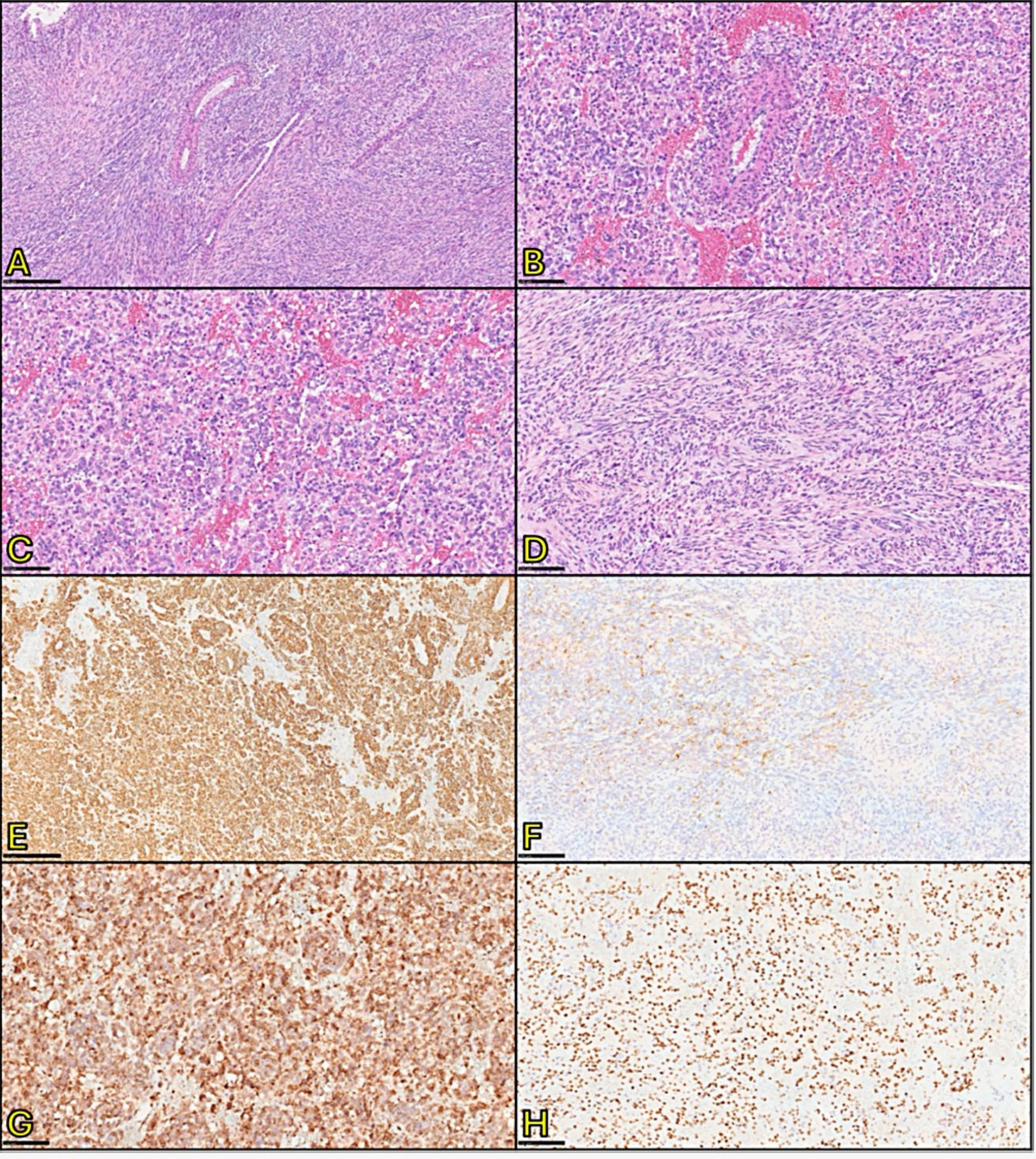
At low magnification, the tumor displays a high cellular density, with neoplastic cells arranged around prominent vascular structures (**A**-**B**, scale bar 250 and 100 μm). At higher magnification, the cells exhibit a dual morphology, with both epithelioid-rhabdoid (**C**, scale bar 100 μm) and spindle-shaped (**D**, scale bar 100 μm) features. Immunohistochemical analysis demonstrated strong SMA expression (**E**, scale bar 250 μm), focal reactivity for HMB45 (**F**, scale bar 100 μm), and diffuse granular cytoplasmic staining for cathepsin K (**G**, scale bar 100 μm); moreover, ER showed nuclear immunoreactivity in a subset of tumor cells (**H**, scale bar 100 μm)

Histopathology demonstrated the classic features of a PEComa: nests and sheets of epithelioid tumor cells arranged around a rich vasculature, with a minor component of rhabdoid and spindle cells (Hu et al. 2024, Bao et al. 2019). The cells had clear to eosinophilic cytoplasm and exhibited marked nuclear atypia, with mitotic figures. Scattered foci of coagulative necrosis were present, fulfilling multiple Folpe criteria for malignancy (tumor > 5 cm, high-grade atypia, mitoses > 1/50 high power fields (HPFs), and necrosis were noted in this case) (Fig. 2) (Folpe et al. 2005). These adverse features correlate with a high malignant potential (Hu et al. 2024, Folpe et al. 2005, Bleeker et al. 2012).

Immunohistochemistry (IHC) was crucial to confirming the diagnosis. The tumor cells showed strong cytoplasmic positivity for HMB-45 and Melan-A, confirming melanocytic differentiation, and co-expressed SMA and desmin, confirming myogenic differentiation. Catepsin K was diffusely positive. This immunoprofile is diagnostic for PEComa (Fig. 2) (Hu et al. 2024, Bao et al. 2019, Bleeker et al. 2012). Other markers aided in the differential diagnosis: pan-cytokeratin and S-100 were negative, ruling out carcinoma, melanoma or peripheral nerve sheath tumor; CD10 and ciclin D1 were also negative, arguing against an endometrial stromal sarcoma; ER and PR showed patchy nuclear staining, which is uncharacteristic of typical leiomyomas. The Ki-67 proliferation index was up to 30%, consistent with a highly proliferative neoplasm (Hu et al. 2024, Bleeker et al. 2012). Notably, the TFE3 immunostain was negative in our case, suggesting no TFE3 gene fusion, in line with most uterine PEComasc (Schoolmeester et al. 2015, Marletta et al. 2025). Overall, histology and IHC firmly established the diagnosis of a malignant uterine PEComa. According to Folpe’s original criteria, our case would be classified as malignant due to the presence of multiple risk features (Folpe et al. 2005). Using more recent uterine-specific schemes, it likewise meets thresholds for high-risk behavior (e.g. Bennett criteria: ≥3 features present) (Bennett et al. 2018).

A comprehensive staging workup was done, given the malignant nature of the tumor. No ovarian involvement was found on pathology. Postoperatively, cross-sectional imaging (chest and abdomen CT) was obtained to search for distant metastases. The lungs are the most common site of metastasis for uterine PEComas, but the patient’s chest CT was clear of any nodules (Sui et al. 2022). No hepatic lesions were seen. Thus, the disease was apparently confined to the uterus: the final stage was pT1b (FIGO stage IB). No adjuvant chemo or radiotherapy was given, in line with the literature (Oliver-Perez et al. 2025, Czarnecka et al. 2023, Sobiborowicz et al. 2020). The patient remains under close surveillance. Thus far, at 24 months of follow-up, she is without any signs of recurrence or metastasis. Surveillance has included periodic physical exams, vaginal cuff cytology, and imaging (semiannual chest and abdominal CT scans) to monitor for relapse. Given that PEComa recurrences often occur within the first 1–2 years (median time ~ 9–12 months in reported series), our patient’s course to date is encouraging (Oliver-Perez et al. 2025). However, long-term follow-up is warranted; late recurrences have been documented, including isolated reports of metastasis arising many years after initial treatment (Hu et al. 2024, Sobiborowicz et al. 2021). We have advised continued imaging surveillance up to at least 5 years postoperatively, with a focus on the lungs due to their predilection as metastatic site. The patient has been counseled about symptoms of recurrence (cough, new pulmonary symptoms, pelvic pain, etc.) and the importance of prompt reporting.

## Discussion

### Epidemiology

PEComas represent a rare and heterogeneous group of mesenchymal neoplasms with variable biological behavior but considered as tumor of uncertain malignant potential. Among these, malignant uterine PEComas are exceedingly uncommon, with few cases reported in the literature to date; consequently, epidemiological data remain limited (Czarnecka et al. 2023). This case underscores the diagnostic and management challenges of malignant uterine PEComa, and our findings align with trends in literature. Initially, the patient was thought to have a benign fibroid: an understandable misdiagnosis given that PEComas often masquerade as more common uterine tumors. In previously reported cases, misidentification as leiomyoma or leiomyosarcoma pre-surgery is frequent (Hu et al. 2024, Cao and Huang 2022). Bennett et al. (2018) noted that uterine PEComas can morphologically resemble smooth muscle tumors, especially when spindle cell areas are present, further confusing the preoperative diagnosis (Bennett et al. 2018). Our case fits this pattern: only after surgical resection and specific IHC studies the correct diagnosis was made. This reflects a broader reality that definitive diagnosis of PEComa relies on pathology, as there are no reliable clinical, laboratory, or imaging hallmarks to identify these tumors beforehand (Oliver-Perez et al. 2025). Therefore, clinicians should maintain a high suspicion for PEComa in any unusual uterine mass, particularly when solitary. From an epidemiologic perspective, our case contributes to the accumulating but still limited data on uterine PEComas. A recent review collected 55 cases of malignant uterine PEComa and reported an average age at diagnosis of ~ 47 years, consistent with our patient being in her 40s (Hu et al. 2024). Notably, that review found a predominance in Asian patients (60%) among reported cases, though it’s unclear if this reflects a true demographic predilection or reporting bias (Hu et al. 2024). No clear environmental or reproductive risk factors have been identified: these tumors appear to arise sporadically, except for the minority associated with TSC.

### Pathogenesis

The origin and pathogenesis of malignant uterine PEComa remain uncertain. Proposed cells of origin include neural crest cells, smooth muscle cells, and pericytic cells, as suggested by pathological and molecular observations, although definitive experimental evidence is still lacking (Gammill and Bronner-Fraser 2003, Stone et al. 2001, Shen et al. 2015).

TSC-associated PEComas often present as multifocal lesions and may exhibit more aggressive behavior (Oliver-Perez et al. 2025, Garzon et al. 2024, Kinzel et al. 2024). The *TSC1*/*TSC2* gene mutations seen in these patients lead to dysregulated mTOR pathway activation, which is a key driver of PEComa growth (Garzon et al. 2024). In contrast, a smaller subset of uterine PEComas is driven by gene fusions involving *TFE3* (a transcription factor), or rarely *RAD51B*, defining a distinct molecular subtype (Schoolmeester et al. 2015, Marletta et al. 2025). These *TFE3*-rearranged PEComas typically lack TSC mutations and may show lower response to mTOR inhibitor therapy (Oliver-Perez et al. 2025, Schoolmeester et al. 2015, Wagner et al. 2021). Overall, molecular pathogenesis points to mTOR signaling dysregulation in the majority of cases, either via *TSC1*/*TSC2* inactivation or alternative gene fusions (Oliver-Perez et al. 2025).

Our patient did not have TSC clinically; only around 8–10% of gynecologic PEComa patients present it (Oliver-Perez et al. 2025). Even so, we pursued *TSC1*/*TSC2* genetic testing given the implication for targeted therapy. While her results were negative, it is noteworthy that 14–15% of uterine PEComas may exhibit somatic *TSC1*/*TSC2* mutations, implying a subset of PEComas may still benefit from mTOR inhibitors even without overt TSC syndrome (Bennett et al. 2022). Another small subset of uterine PEComas is driven by *TFE3* gene fusions: these tend to occur in younger patients and can lack muscle marker expression (Marletta et al. 2025). Recognizing such molecular differences is important, as *TFE3*-rearranged PEComas may respond less favorably to mTOR inhibition and could warrant alternative strategies (e.g. exploration of MET inhibitors).

### Diagnosis

Clinically, uterine PEComas pose a significant diagnostic challenge due to non-specific presentations. Patients most often report abnormal uterine bleeding or pelvic pain, indistinguishable from far more common uterine neoplasms (Oliver-Perez et al. 2025). After reviewing the literature, it emerged that 41.8% of patients present with vaginal bleeding and 25.4% with pelvic or abdominal pain (Musella et al. 2015, Oliver-Perez et al. 2025, Hu et al. 2024, Conlon et al. 2015, Schoolmeester et al. 2014, Wang et al. 2024, Fontana et al. 2021, Phillips et al. 2016, Bennett et al. 2018). Less commonly, a pelvic mass is palpable on exam, or the tumor is discovered incidentally on imaging or surgery (Conlon et al. 2015, Schoolmeester et al. 2014). Rare acute presentations (such as uterine rupture or hemoperitoneum) have been reported, usually with large, fast-growing tumors or in pregnancy (Nguyen et al. 2020). No specific serum tumor markers are associated with PEComas; for instance, CA-125 is occasionally elevated but is nonspecific (Sui et al. 2022). Preoperative imaging features are also non-diagnostic: ultrasound, CT, and MRI typically show a well-circumscribed uterine mass that may contain areas of hemorrhage, necrosis, or even fat, but these findings overlap with benign fibroids or uterine sarcomas (Phillips et al. 2016). Notably, on ultrasound these tumors often appear heterogeneous with a rich central vascular network and indistinct borders (Wang et al. 2024, Fontana et al. 2021). Such features frequently lead to initial misdiagnosis as a leiomyoma or other stromal tumor (Wang et al. 2024). Indeed, most reported uterine PEComa cases were not correctly identified until histopathological examination after surgery (Wang et al. 2024, Fontana et al. 2021, Phillips et al. 2016). This underlies the importance of a high index of suspicion and thorough pathologic evaluation for any unusual uterine mass. Lesions most frequently arise in the uterine corpus, with less common involvement of the cervix, broad ligament, or round ligament (Hu et al. 2024).

### Histology

From a histologic perspective, PEComas are defined by perivascular epithelioid cells, typically epithelioid-shaped tumor cells with clear to granular eosinophilic cytoplasm arranged around blood vessels in nests or sheets (Oliver-Perez et al. 2025, Conlon et al. 2015). Many tumors also contain a spindle cell component, and cells can show varying degrees of pleomorphism (Conlon et al. 2015). By IHC, uterine PEComas almost invariably co-express melanocytic markers (HMB-45 is most specific) and muscle markers (SMA, desmin) (Bennett et al. 2022). Melan-A and MiTF are also positive in a majority of cases, albeit more focally (Bennett et al. 2022). This dual immunoprofile is a diagnostic hallmark that helps distinguish PEComas from other uterine neoplasms: for example, uterine smooth muscle tumors lack melanocytic marker expression. Strong and diffuse nuclear immunopositivity for TFE3 can be observed in tumors harboring TFE3 gene rearrangements, a subset that may conversely exhibit reduced expression of muscle markers. However, the sensitivity and specificity of TFE3 immunohistochemistry depend on the type of antibody used (polyclonal vs. monoclonal), as well as on tissue fixation and staining conditions. Therefore, while TFE3 can serve as a useful screening tool, its limited specificity necessitates confirmatory molecular studies, given that low-level expression is also observed physiologically in a wide range of normal tissues. Additional markers like Cathepsin K have high sensitivity for PEComa and are frequently positive (Martignoni et al. 2012). Uterine PEComas are typically negative for lineage specific markers, though focal ER/PR, S-100 and cytokeratins positivity can be observed in some tumors (Hu et al. 2024, Armah and Parwani 2007). The main differential diagnoses are leiomyoma, leiomyosarcoma, endometrial stromal sarcoma and undifferentiated uterine sarcoma.

As already said, according to the Folpe et al. criteria, features supporting malignancy in PEComa include size > 5 cm, infiltrative growth pattern, high nuclear grade, mitotic rate > 1/50 HPFs, necrosis, and vascular invasion (Folpe et al. 2005). At least three of these features are required for a diagnosis of malignant uterine PEComa. In our case, the tumor measured 9 cm, exhibited high nuclear grade as well as scattered foci of necrosis, and demonstrated a mitotic index above the defined threshold, thereby fulfilling the histological criteria for malignancy.

However, subsequent studies noted that Folpe’s original scheme, derived from only 4 uterine cases out of 26 total, has limited predictive accuracy for gynecologic PEComas (Jiang et al. 2023, Conlon et al. 2015). Refinements have since been proposed: modified Folpe criteria treat any single risk factor as indeterminate rather than frankly malignant, requiring two or more for malignancy; Schoolmeester’s criteria (based on metastatic uterine PEComas) suggested that only tumors with ≥ 4 worrisome features be deemed malignant, while Bennett’s criteria define malignancy by ≥ 3 features present (Conlon et al. 2015, Schoolmeester et al. 2014, Bennett et al. 2018). These classification systems each aim to improve specificity, though no consensus exists yet. Notably, one comparative analysis found the modified Folpe system had the best correlation with actual outcomes in uterine PEComas (Garzon et al. 2024). In practice, any uterine PEComa showing multiple adverse histologic features is approached as malignant, given the substantial risk of recurrence.

### Treatment and future directions

Our patient’s treatment course highlights the current management paradigm for malignant PEComa: surgical resection followed by case-by-case consideration of adjuvant therapy. Complete surgical excision with clear margins is the cornerstone of treatment for localized PEComa (Sobiborowicz et al. 2020). In gynecologic cases, this usually means total hysterectomy (with or without oophorectomy), as was performed in our case. Lymphadenectomy is not routinely recommended unless nodes are enlarged, since lymphatic spread is relatively uncommon (Czarnecka et al. 2023). Regarding the ovaries, some authors advocate salpingo-oophorectomy, especially if the tumor is high-grade or involves the uterine serosa/adnexa, to eliminate potential metastatic sites (Musella et al. 2015, Cui et al. 2024). We elected to remove the ovaries in our patient, given the tumor’s aggressive features, though ovarian metastases from uterine PEComa are rare (about < 5–10% of cases), so fertility preservation may be considered in low-risk, selected patients (Oliver-Perez et al. 2025).

The role of adjuvant therapy in uterine PEComa remains unclear due to the absence of clinical trials or guidelines. Traditional cytotoxic chemotherapy (e.g. doxorubicin, ifosfamide, or gemcitabine-based regimens) has been used in some reported cases, but PEComas are generally considered chemoresistant with only modest responses (Czarnecka et al. 2023, Sobiborowicz et al. 2020). In the literature, no clear survival benefit for adjuvant chemo was demonstrable, and thus some experts reserve it for metastatic or unresectable disease (Czarnecka et al. 2023, Sobiborowicz et al. 2020, Sobiborowicz et al. 2021).

Increasingly, molecular targeted therapy is at the forefront of managing advanced PEComas. The mTOR pathway is a logical target due to *TSC1-2* gene mutations; multiple case reports and small series have documented meaningful tumor regressions with mTOR inhibitors like sirolimus, everolimus, or temsirolimus (Sui et al. 2022, Sobiborowicz et al. 2021, Wagner et al. 2021, Gennatas et al. 2012, Benson et al. 2014). The pivotal AMPECT trial demonstrated a nearly 40% response rate to nab-sirolimus (ABI-009) in metastatic PEComa, leading to its FDA approval (Wagner et al. 2024). While our patient did not receive mTOR therapy, this option may be preserved for any future recurrence. For patients who progress on mTOR inhibitors, other strategies reported include combining mTOR inhibitors with VEGFR tyrosine kinase inhibitors (e.g. concurrent sirolimus and sunitinib) to overcome resistance (Gao et al. 2016). There are also instances of hormonal therapy (e.g. progesterone or aromatase inhibitors) being tried if the tumor expresses hormone receptors, sometimes in combination with mTOR blockade, although evidence of benefit is limited (Sanfilippo et al. 2019, Le et al. 2014). Anti-angiogenic agents alone (pazopanib, sorafenib, etc.) have modest activity and are generally second-line in advanced PEComa (Liapi et al. 2021). Immunotherapy is largely experimental in this disease, but anecdotal successes with checkpoint inhibitors (especially in TFE3-fusion cases that may have a different biology) have been noted in case reports (Rémond et al. 2024).

### Outcomes

In terms of prognosis, malignant PEComas of the uterus carry a substantial risk of recurrence, yet not all behave aggressively. Our patient remains disease-free at 2 years, aligning with the roughly 60–70% of patients who do not develop recurrence in short-term follow-up (Oliver-Perez et al. 2025, Hu et al. 2024). However, published series caution that recurrence rates around 30–35% are observed, often within the first year after therapy (Garzon et al. 2024). Jiang et al. reported a 31% recurrence rate and ~ 6.5% disease-specific mortality at 2 years median follow-up, highlighting that early relapses are not uncommon (Jiang et al. 2023). For those who recur or metastasize, long-term survival is variable; some achieve prolonged remission with surgery and mTOR therapy, while others have progressive disease refractory to treatment. Our patient’s case also illustrates the need for long-term surveillance. Given the possibility of late recurrence, we plan ongoing annual imaging after the initial intensive 2-year surveillance period. There are no formal guidelines, but many experts follow these patients similarly to high-grade uterine sarcomas, with frequent monitoring in the first 2–3 years and continued follow-up up to 5–10 years post-treatment.

## Conclusion

In conclusion, malignant uterine PEComa is rare but poses important diagnostic challenges for uterine mesenchymal tumors. Our narrative review, together with this rare and challenging case report, exemplifies the typical scenario of misdiagnosis and subsequent surprise pathology diagnosis, the application of Folpe’s criteria to recognize malignancy, and the multidisciplinary approach needed. The literature review reveals that while surgery remains the cornerstone, adjunct therapies like mTOR inhibitors are emerging as valuable tools in the management of advanced or recurrent PEComas. The prognosis is guarded for high-risk PEComas, but with vigilant follow-up and modern therapies, patients like ours can achieve meaningful disease control. Continued documentation of cases and, possibly, multicentric studies are needed to refine risk stratification and guide adjuvant treatment for this rare gynecologic tumor. Each new case report, such as this one, adds to our understanding and helps build evidence for managing future patients with this challenging diagnosis.

## Data Availability

The authors declare that all the data hereby presented are included in the manuscript and/or its supplementary material.

## References

[CR35] Armah HB, Parwani AV (2007) Malignant perivascular epithelioid cell tumor (PEComa) of the uterus with late renal and pulmonary metastases: a case report with review of the literature. Diagn Pathol 2:45. 10.1186/1746-1596-2-45 18053181 10.1186/1746-1596-2-45 PMC2213634

[CR17] Bao L, Shi Y, Zhong J, Zhao M, Wu J, Hai L, Xu X, Du H, Shi Y (2019) Histopathologic characteristics and immunotypes of perivascular epithelioid cell tumors (PEComa). Int J Clin Exp Pathol 12(12):4380–4389 31933841 PMC6949869

[CR22] Bennett JA, Braga AC, Pinto A, Van de Vijver K, Cornejo K, Pesci A, Zhang L, Morales-Oyarvide V, Kiyokawa T, Zannoni GF, Carlson J, Slavik T, Tornos C, Antonescu CR, Oliva E (2018) Uterine PEComas: a morphologic, immunohistochemical, and molecular analysis of 32 tumors. Am J Surg Pathol 42(10):1370–1383. 10.1097/PAS.0000000000001119 30001237 10.1097/PAS.0000000000001119 PMC6133752

[CR33] Bennett JA, Ordulu Z, Pinto A, Wanjari P, Antonescu CR, Ritterhouse LL, Oliva E (2022) Uterine PEComas: correlation between melanocytic marker expression and TSC alterations/TFE3 fusions. Mod Pathol 35(4):515–523. 10.1038/s41379-021-00855-1 34131293 10.1038/s41379-021-00855-1 PMC8671557

[CR38] Benson C, Vitfell-Rasmussen J, Maruzzo M, Fisher C, Tunariu N, Mitchell S, Al-Muderis O, Thway K, Larkin J, Judson I (2014) A retrospective study of patients with malignant PEComa receiving treatment with sirolimus or temsirolimus: the Royal Marsden Hospital experience. Anticancer Res 34(7):3663–3668 24982384

[CR19] Bleeker JS, Quevedo JF, Folpe AL (2012) Malignant perivascular epithelioid cell neoplasm: risk stratification and treatment strategies. Sarcoma 2012:541626. 10.1155/2012/541626 22619565 10.1155/2012/541626 PMC3350998

[CR15] Cao B, Huang Y (2022) Malignant perivascular epithelioid cell tumor (PEComa) of the uterus. BMC Womens Health 22(1):523. 10.1186/s12905-022-02119-9 36522714 10.1186/s12905-022-02119-9 PMC9756506

[CR6] Conlon N, Soslow RA, Murali R (2015) Perivascular epithelioid tumours (PEComas) of the gynaecological tract. J Clin Pathol 68(6):418–426. 10.1136/jclinpath-2015-202945 25750268 10.1136/jclinpath-2015-202945 PMC4984252

[CR36] Cui Q, Li C, Huang T, Huang J, Chen M (2024) Systematic analysis of perivascular epithelioid cell neoplasms in the female reproductive tract: a comprehensive review. Future Oncol 20:283–295 38426361 10.2217/fon-2023-0778

[CR24] Czarnecka AM, Skoczylas J, Bartnik E, Świtaj T, Rutkowski P (2023) Management strategies for adults with locally advanced, unresectable or metastatic malignant perivascular epithelioid cell tumor (PEComa): challenges and solutions. Cancer Manag Res 15:615–623. 10.2147/CMAR.S351284 37440783 10.2147/CMAR.S351284 PMC10335286

[CR7] Fadare O (2008) Uterine PEComa: appraisal of a controversial and increasingly reported mesenchymal neoplasm. Int Semin Surg Oncol 5:7. 10.1186/1477-7800-5-7 18325099 10.1186/1477-7800-5-7 PMC2278149

[CR1] Fink D, Marsden DE, Edwards L, Camaris C, Hacker NF (2004) Malignant perivascular epithelioid cell tumor (PEComa) arising in the broad ligament. Int J Gynecol Cancer 14(5):1036–1039. 10.1111/j.1048-891X.2004.014549.x 15361222 10.1111/j.1048-891X.2004.014549.x

[CR18] Folpe AL, Mentzel T, Lehr HA, Fisher C, Balzer BL, Weiss SW (2005) Perivascular epithelioid cell neoplasms of soft tissue and gynecologic origin: a clinicopathologic study of 26 cases and review of the literature. Am J Surg Pathol 29(12):1558–1575. 10.1097/01.pas.0000173232.22117.37 16327428 10.1097/01.pas.0000173232.22117.37

[CR11] Fontana E, Savelli L, Alletto A, Seracchioli R (2021) Uterine PEComa initially misdiagnosed as a leiomyoma: sonographic findings and review of the literature. J Clin Ultrasound 49(5):492–497. 10.1002/jcu.22950 33197067 10.1002/jcu.22950

[CR13] Gagnier JJ, Kienle G, Altman DG, Moher D, Sox H, Riley D, CARE Group (2013) The CARE guidelines: consensus-based clinical case reporting guideline development. Glob Adv Health Med 2(5):38–43. 10.7453/gahmj.2013.008 24416692 10.7453/gahmj.2013.008 PMC3833570

[CR27] Gammill LS, Bronner-Fraser M (2003) Neural crest specification: migrating into genomics. Nat Rev Neurosci 4(10):795–805. 10.1038/nrn1219 14523379 10.1038/nrn1219

[CR40] Gao F, Huang C, Zhang Y, Sun R, Zhang Y, Wang H, Zhang S (2016) Combination targeted therapy of VEGFR inhibitor sorafenib with an mTOR inhibitor sirolimus induced a remarkable response of rapid progressive uterine PEComa. Cancer Biol Ther 17:595–598 27030639 10.1080/15384047.2016.1167290 PMC4990405

[CR30] Garzon S, Caliò A, Ferrari FA, Iannicello CQ, Zorzato PC, Bosco M, Piazzola E, Martignoni G, Laganà AS, Mariani A et al (2024) Uterine perivascular epithelioid cell tumors (PEComa) and the accuracy of proposed classification systems in predicting malignant versus non-malignant behavior. Gynecol Oncol 188:35–43. 10.1016/j.ygyno.2024.06.007 38905754 10.1016/j.ygyno.2024.06.007

[CR37] Gennatas C, Michalaki V, Kairi PV, Kondi-Paphiti A, Voros D (2012) Successful treatment with the mTOR inhibitor everolimus in a patient with perivascular epithelioid cell tumor. World J Surg Oncol 10:181. 10.1186/1477-7819-10-181 22943457 10.1186/1477-7819-10-181 PMC3499435

[CR14] Giannella L, Delli Carpini G, Montik N, Verdecchia V, Puccio F, Di Giuseppe J, Tsiroglou D, Goteri G, Ciavattini A (2020) Ultrasound features of a uterine perivascular epithelioid cell tumor (PEComa): case report and literature review. Diagnostics (Basel) 10(8):553. 10.3390/diagnostics10080553 32756336 10.3390/diagnostics10080553 PMC7459969

[CR4] Hu D, Miao M, Zhou H, Gu X, Wang X, Teichmann AT, Wang Q, Yang Y (2024) A case report of malignant perivascular epithelioid cell tumors of the uterus and literature review. Int J Womens Health 16:619–628. 10.2147/IJWH.S453226 38645980 10.2147/IJWH.S453226 PMC11027917

[CR5] Jiang Y, Liu X, Zhang S, Wang Q, Xu Q, Ghias K, Cao L (2023) Risk stratification and outcomes in 210 gynecologic perivascular epithelioid cell tumors (PEComas) cases. Arch Gynecol Obstet 307:681–687 35411411 10.1007/s00404-022-06470-y

[CR31] Kinzel A, McArthur M, Gettle LM, Felker E, Patel M (2024) PEComas: a review of imaging and clinical features. Clin Imaging 116:110332. 10.1016/j.clinimag.2024.110332 39442258 10.1016/j.clinimag.2024.110332

[CR42] Le P, Garg A, Brandao G, Abu-Sanad A, Panasci L (2014) Hormonal manipulation with letrozole in the treatment of metastatic malignant PEComa. Curr Oncol 21:e518–e520 24940112 10.3747/co.21.1849 PMC4059816

[CR43] Liapi A, Mathevet P, Herrera FG, Hastir D, Sarivalasis A (2021) VEGFR inhibitors for uterine metastatic perivascular epithelioid tumors (PEComa) resistant to mTOR inhibitors: case report and review of the literature. Front Oncol 11:641376. 10.3389/fonc.2021.641376 33842348 10.3389/fonc.2021.641376 PMC8032946

[CR21] Marletta S, Caliò A, Pierconti F, Harada S, Netto GJ, Antonini P, Segala D, Pedron S, Marcolini L, Stefanizzi L, Martignoni G (2025) SFPQ::TFE3-rearranged PEComa: differences and analogies with renal cell carcinoma carrying the same translocation. Pathol Res Pract 270:155963. 10.1016/j.prp.2025.155963 40239600 10.1016/j.prp.2025.155963

[CR34] Martignoni G, Bonetti F, Chilosi M, Brunelli M, Segala D, Amin MB, Argani P, Eble JN, Gobbo S, Pea M (2012) Cathepsin K expression in the spectrum of perivascular epithelioid cell (PEC) lesions of the kidney. Mod Pathol 25(1):100–111. 10.1038/modpathol.2011.136 21874011 10.1038/modpathol.2011.136

[CR2] Musella A, De Felice F, Kyriacou AK, Barletta F, Di Matteo FM, Marchetti C, Izzo L, Monti M, Benedetti Panici P, Redler A, D’Andrea V (2015) Perivascular epithelioid cell neoplasm (PEComa) of the uterus: a systematic review. Int J Surg 19:1–5. 10.1016/j.ijsu.2015.05.002 25981307 10.1016/j.ijsu.2015.05.002

[CR9] Nguyen JMV, Ghandehari H, Parra-Herran C, Vicus D (2020) Uterine rupture: an unusual presentation of a uterine perivascular epithelioid cell tumor (PEComa). Int J Gynecol Cancer 30(12):2008–2011. 10.1136/ijgc-2020-001837 32763950 10.1136/ijgc-2020-001837

[CR3] Oliver-Perez R, Ortega M, Manzano A, Estrada-Lorenzo JM, Martinez-Lopez M, Zabia E, Lopez-Gonzalez G, Madariaga A, Parrilla L, Tejerizo A et al (2025) Malignant perivascular epithelioid cell tumor (PEComa) of the uterus: a rare type of mesenchymal tumors and a management challenge. Cancers (Basel) 17:2185. 10.3390/cancers17132185 40647483 10.3390/cancers17132185 PMC12249296

[CR12] Phillips CH, Keraliya AR, Shinagare AB, Ramaiya NH, Tirumani SH (2016) Update on the imaging of malignant perivascular epithelioid cell tumors (PEComas). Abdom Radiol (NY) 41(2):368–376. 10.1007/s00261-015-0568-8 26867923 10.1007/s00261-015-0568-8

[CR44] Rémond M, Pachev A, Battistella M, Gandon C, Mourah S, Madelaine I, Maggiori L, Benadon B, Hammoudi N, Lourenço N, Aparicio T (2024) Metastatic perirectal PEComa treated by checkpoint inhibitor immunotherapy and multimodal treatment: case report and review of the literature. Ther Adv Med Oncol 16:17588359241280541. 10.1177/17588359241280541 39314916 10.1177/17588359241280541 PMC11418325

[CR41] Sanfilippo R, Jones RL, Blay JY, Le Cesne A, Provenzano S, Antoniou G, Mir O, Fucà G, Fumagalli E, Bertulli R et al (2019) Role of chemotherapy, VEGFR inhibitors, and mTOR inhibitors in advanced perivascular epithelioid cell tumors (PEComas). Clin Cancer Res 25:5295–5300 31217199 10.1158/1078-0432.CCR-19-0288

[CR20] Schoolmeester JK, Dao LN, Sukov WR, Wang L, Park KJ, Murali R, Hameed MR, Soslow RA (2015) TFE3 translocation-associated perivascular epithelioid cell neoplasm (PEComa) of the gynecologic tract: morphology, immunophenotype, differential diagnosis. Am J Surg Pathol 39(3):394–404. 10.1097/PAS.0000000000000349 25517951 10.1097/PAS.0000000000000349 PMC4982474

[CR8] Schoolmeester JK, Howitt BE, Hirsch MS, Dal Cin P, Quade BJ, Nucci MR (2014) Perivascular epithelioid cell neoplasm (PEComa) of the gynecologic tract: clinicopathologic and immunohistochemical characterization of 16 cases. Am J Surg Pathol 38(2):176–188. 10.1097/PAS.0000000000000133 24418852 10.1097/PAS.0000000000000133

[CR29] Shen J, Shrestha S, Yen YH, Scott MA, Asatrian G, Barnhill R, Lugassy C, Soo C, Ting K, Peault B, Dry SM, James AW (2015) Pericyte antigens in angiomyolipoma and PEComa family tumors. Med Oncol 32(8):210. 10.1007/s12032-015-0659-y 26123600 10.1007/s12032-015-0659-y

[CR25] Sobiborowicz A, Czarnecka AM, Szumera-Ciećkiewicz A, Rutkowski P, Świtaj T (2020) Diagnosis and treatment of malignant PEComa tumours. Oncol Clin Pract 16(1):22–33. 10.5603/OCP.2020.0003

[CR26] Sobiborowicz A, Świtaj T, Teterycz P, Spałek MJ, Szumera-Ciećkiewicz A, Wągrodzki M, Zdzienicki M, Czarnecka AM, Rutkowski P (2021) Feasibility and long-term efficacy of PEComa treatment—20 years of experience. J Clin Med 10(10):2200. 10.3390/jcm10102200 34069629 10.3390/jcm10102200 PMC8160690

[CR28] Stone CH, Lee MW, Amin MB, Yaziji H, Gown AM, Ro JY, Têtu B, Paraf F, Zarbo RJ (2001) Renal angiomyolipoma: further immunophenotypic characterization of an expanding morphologic spectrum. Arch Pathol Lab Med 125(6):751–758. 10.5858/2001-125-0751-RA 11371226 10.5858/2001-125-0751-RA

[CR23] Sui C, Wu J, Mei D, Pan E, Yang P, Wu T, Ma Y, Ou Q, Song L (2022) Uterine perivascular epithelioid tumors (PEComas) with lung metastasis showed good responses to mTOR and VEGFR inhibitors: a case report. Front Oncol 12:797275. 10.3389/fonc.2022.797275 35965503 10.3389/fonc.2022.797275 PMC9366196

[CR39] Wagner AJ, Ravi V, Riedel RF, Ganjoo K, Van Tine BA, Chugh R, Cranmer L, Gordon EM, Hornick JL, Du H, Ding L, Schmid AN, Navarro WH, Kwiatkowski DJ, Dickson MA (2024) Phase II trial of nab-sirolimus in patients with advanced malignant perivascular epithelioid cell tumors (AMPECT): long-term efficacy and safety update. J Clin Oncol 42(13):1472–1476. 10.1200/JCO.23.02266 38427923 10.1200/JCO.23.02266 PMC11095855

[CR32] Wagner AJ, Ravi V, Riedel RF, Ganjoo K, Van Tine BA, Chugh R, Cranmer L, Gordon EM, Hornick JL, Du H et al (2021) nab-Sirolimus for patients with malignant perivascular epithelioid cell tumors. J Clin Oncol 39:3660–3670. 10.1200/JCO.21.01728 34637337 10.1200/JCO.21.01728 PMC8601264

[CR10] Wang R, Luo H, Cao W (2024) Clinical and ultrasound features of uterine perivascular epithelioid cell tumors: case series and literature review. Ultrasound Obstet Gynecol 64(5):687–695. 10.1002/uog.29116 39395194 10.1002/uog.29116

[CR16] Yu X, Duan R, Yang B, Huang L, Hou M, Qie M (2024) Perivascular epithelioid cell tumor of the uterus and pelvic cavity. Front Oncol 14:1449936. 10.3389/fonc.2024.1449936 39540153 10.3389/fonc.2024.1449936 PMC11557460

